# Poly(hydroxyurethane) Adhesives and Coatings: State-of-the-Art and Future Directions

**DOI:** 10.1021/acssuschemeng.1c02558

**Published:** 2021-07-14

**Authors:** Alvaro Gomez-Lopez, Satyannarayana Panchireddy, Bruno Grignard, Inigo Calvo, Christine Jerome, Christophe Detrembleur, Haritz Sardon

**Affiliations:** † POLYMAT and Polymer Science and Technology Department, Faculty of Chemistry, University of the Basque Country UPV/EHU, Paseo Manuel de Lardizabal 3, 20018 Donostia-San Sebastián, Spain; ‡ Center for Education and Research on Macromolecules (CERM), CESAM Research Unit, University of Liège, allée du 6 août, Building B6A, Agora Square, 4000 Liège, Belgium; § ORIBAY Group Automotive S.L. R&D Department, Portuetxe bidea 18, 20018 Donostia-San Sebastián, Spain

**Keywords:** Sustainability, Non-isocyanate polyurethane, Adhesive, Coating, Poly(hydroxyurethane), Cyclic carbonate, Polyaddition

## Abstract

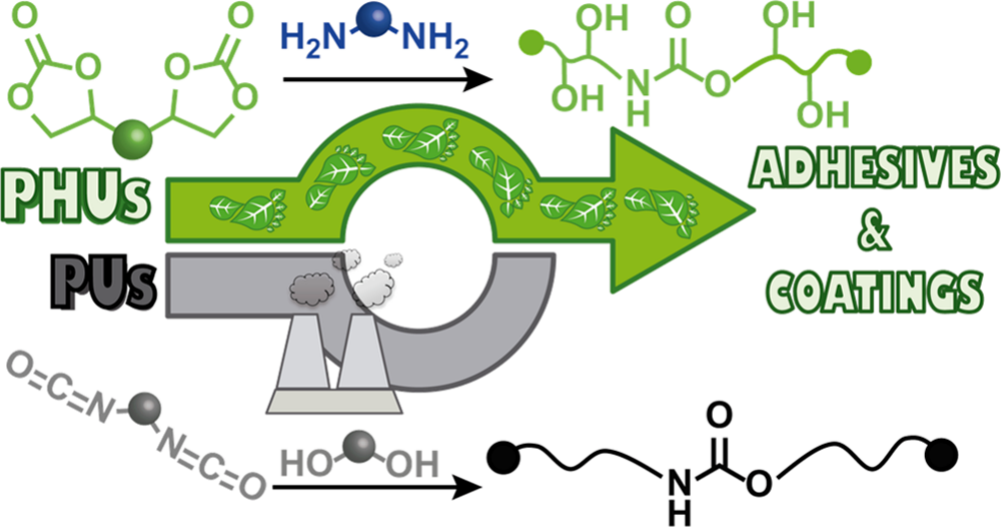

Polyurethane (PU) adhesives and coatings are widely used to fabricate high-quality materials due to their excellent properties and their versatile nature, which stems from the wide range of commercially available polyisocyanate and polyol precursors. This polymer family has traditionally been used in a wide range of adhesive applications including the bonding of footwear soles, bonding of wood (flooring) to concrete (subflooring), in the automotive industry for adhering different car parts, and in rotor blades, in which large surfaces are required to be adhered. Moreover, PUs are also frequently applied as coatings/paints for automotive finishes and can be applied over a wide range of substrates such as wood, metal, plastic, and textiles. One of the major drawbacks of this polymer family lies in the use of toxic isocyanate-based starting materials. In the context of the REACH regulation, which places restrictions on the use of substances containing free isocyanates, it is now urgent to find greener routes to PUs. While non-isocyanate polyurethanes (NIPUs) based on the polyaddition of poly(cyclic carbonate)s to polyamines have emerged in the past decade as greener alternatives to conventional PUs, their industrial implementation is at an early stage of development. In this review article, recent advances in the application of NIPUs in the field of adhesives and coatings are summarized. The article also draws attention to the opportunities and challenges of implementing NIPUs at the industrial scale.

## Abstract 2

Sustainable poly(hydroxyurethane) is explored as a greener alternative to toxic and harmful conventional polyurethanes employed in adhesive and coating applications.

## Introduction

**Figure 1:**
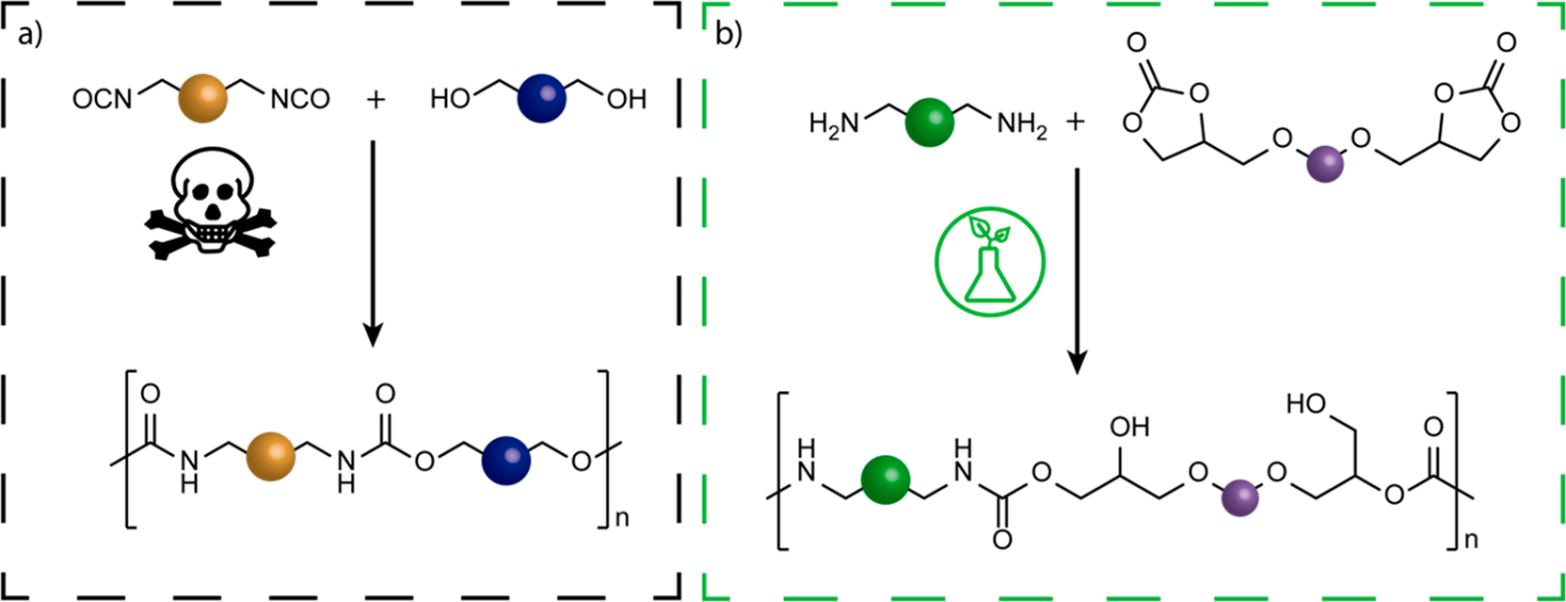
(a) Conventional synthesis of polyurethanes through step-growth polymerization of polyisocyanate and polyol. (b) Synthesis of non-isocyanate polyurethanes by polyaddition of bis(cyclic carbonate) to diamine.

Polyurethanes (PUs) are highly versatile polymers that are widely employed in modern life as rigid or flexible foams, as well as in elastomers, composite materials, paints, coatings, and adhesives. Currently, their annual worldwide production exceeds 20 million tons and accounts for about 7 wt % of all plastic production.^1^ The polyurethane adhesives market size was estimated at 7.0 billion USD in 2019 and is projected to grow to 9.1 million USD by 2024 (at a 5.6% compound annual growth rate).^2^ Similar values are predicted for the polyurethane coatings market, which was estimated at over 18 billion USD in 2020.^3^ The automotive and transportation industry represents the largest consumer/end-user application of polyurethanes in both cases. Adhesives as well as coatings are widely employed in the wood, furniture, building, and construction industries. The packaging and footwear industries also contribute to the adhesives market, while there is a sizable market share for PU coatings used in electronic applications.^2,3^ Nevertheless, since the first patent of Bayer^4^ in 1937, the chemistry behind PUs has remained largely unchanged, and the majority of commercially available PUs are fabricated by the step-growth copolymerization of polyisocyanates and polyols (Figure 1a). The physicochemical properties of PUs are largely guided by the nature, the stoichiometry, and/or the functionality of the monomers, while their unique thermomechanical behavior depends on the intrinsic tendency of the chains to phase segregate due to the strong hydrogen bonds between urethane moieties. On account of their diverse formulation options, PUs are the preferred choice when it comes to formulating high-quality coatings or adhesives with excellent adhesion, resistance to abrasion, chemical resistance, and low temperature tolerance.^5^

Despite the good performance of PUs in a wide range of applications, the inherent toxicity of the isocyanates, which are known to cause asthma and dermatitis,^6−8^ as well as the chemicals used in their production, most notably the use of phosgene,^9^ are major drawbacks of this chemistry. In this context, the REACH regulation now restricts the use of isocyanates, and the quest for new, safer, and greener alternatives to isocyanates is becoming one of the major challenges in the PU industry. Thus, in the past decade non-isocyanate polyurethanes (NIPUs) have emerged as alternatives in the search for greener PUs (Figure 1b).

Moreover, the vast majority of industrially applied PUs are based on non-sustainable feedstocks including crude oil and gas. Therefore, there is a drive to not only limit the use of isocyanates but also substitute fossil resources with renewable ones in order to move toward a more sustainable industry.^10^

Various routes have been developed to synthesize NIPUs from a diverse range building blocks such as (i) the ring-opening polymerization of carbamates, (ii) the copolymerization of aziridines with CO_2_, (iii) the polycondensation of bis(dialkylcarbonate)s with diamines or bis(dialkylcarbamate)s with diols, and (iv) the polyaddition of poly(cyclic carbonate)s to polyamines. Of these, the latter pathway is by far the most popular and competitive approach.^11,12^ Indeed, this polyaddition provides poly(hydroxyurethane)s (PHUs) based on a 100% atom economy approach and a large portfolio of poly(cyclic carbonate)s is now easily accessible at low cost from multiple chemistries. The most popular is the facile chemical [3 + 2] CO_2_ insertion into their corresponding (biobased) epoxy precursors^13−17^ providing materials with reduced/low carbon footprint, in line with the sustainability requirements of our society. Unlike isocyanates, cyclic carbonates are much less sensitive to moisture, enabling their facile long-term storage and manipulation.^18^

In view of the increasing developments in the field of NIPU synthesis and applications, this review aims at providing a critical view of the current state-of-the-art of these polymers in adhesives and coatings with a focus on the open literature and patents. The characteristics of conventional PU adhesives and coatings will be briefly introduced to illustrate the scope of their application and to provide the guidelines to construct the next generation of NIPU adhesives and coatings with equal, or even superior, performance. The last part of the review will provide some general conclusions and future perspectives for the use of NIPUs and will focus on the main obstacles and potential solutions that might facilitate the transfer of the technology to industry.

## PU Adhesives and Coatings: Main Processes and Characterization Tools

### Main Processing Methods and Applications of PU Adhesives and Coatings

To design adhesives and coatings that compete with the performance of conventional PU materials, the formulation and processing of NIPUs should conform to certain characteristics. This necessitates the identification of the key features of conventional PU materials and the understanding of how they affect the final application. To fabricate PU adhesives that display an optimal balance between adhesive and cohesive forces, most systems are chemically cross-linked. Aromatic isocyanates are also preferred as they possess a uniform reactivity of reactive groups, lower volatility resulting in easier workplace handling, and lower prices than aliphatic isocyanates. Polyether polyols are usually chosen as comonomers as they offer improved low-temperature flexibility, are in a liquid state with acceptable viscosity at room temperature, and are much less sensitive to hydrolysis than polyesters.^19^ Adhesives can be classified as either chemical reactive formulations, thermoplastics, or evaporation systems. Chemical reactive adhesives include two component systems and moisture-, heat-, or UV-sensitive groups. Usually these are supplied in a low molar mass form and polymerization occurs after application. Thermoplastics are basically hot-melt technologies, in which the adhesive flows at elevated temperature and solidifies when the temperature is decreased below their *T*_g_ or *T*_m_. In evaporation or diffusion type adhesives, polymers are applied in their final form, either dissolved or dispersed in a suitable solvent. In terms of sustainability, waterborne formulations are preferred vs solvent-based ones. To date, the formulation and processing of polyurethane adhesives differ regarding the envisioned application and correspond to one of the five technologies summarized in Table 1, each of which is suited for specific applications.

**Table 1:** General Processing Methods and Applications of PU Adhesives and Coatings

technology	description	applications
solvent-free	1K systems usually silane-terminated prepolymers with good adhesion to glass	1K are typically used in automotive industry while 2K are more employed in the building sector or flooring applications
	2K systems are applied when rapid curing is needed and for structural adhesives	
hot-melt	reactive systems bearing a low percentage of free isocyanate which reacts with air moisture or functional moieties on the surface of the substrates	wood industry or shoe soles
solvent-based	high-molar mass prepolymers prepared with a slight excess of NCO groups	shoes, food packaging, automotive, and furniture industry
water-borne	ionizable moiety is present to allow the dispersion of 1K as well as 2K formulations based on temperature sensitive reactive systems	foot wear, bookbinding, furniture, textile laminates
radiation curable	acrylate-tipped prepolymers endowing the adhesives with faster curing and higher stability	flexible and heat-sensitive substrates

PU coatings are similar in nature to adhesives as, in both cases, a viscous reactive formulation must be applied onto a substrate, the surface must be wet, and a homogeneous film must be formed and must adhere to the surface. Nevertheless, for coatings, the chemical resistance, flexibility, and appearance of the final product are more important, while bond strength and shear resistance are not as relevant as in adhesives. Table 2 shows an ASTM convention to classify PU coatings according to their general characteristics and the possible scope of their applications. Similar to adhesives, some general statements can be made with regard to the formulation of PU coatings including the following: (a) aliphatic isocyanates are the preferred choice for decorative coatings as they provide enhanced UV-light resistance, while aromatic isocyanates are employed in nondecorative applications and (b) acrylic polyols are commonly used as soft segments due to the tough and high resistance of the resulting coatings.^19^

**Table 2:** ASTM Convention for a PU Coating Classification, Its Technology Definition, and Typical Applications, Adapted from the Work of Sonnenschein^19^

ASTM convention	technology	applications
type I	cured by oxidative cross-linking of unsaturated polyester groups and solvent evaporation	architectural floors and maintenance, topcoats
type II	contains free isocyanates, reacts with moisture; various blocking techniques to preserve isocyanate reactivity for extended shelf life	leathers, concretes, maintenance
type III	one-part heat cure; uses blocked isocyanates that are liberated upon heating to react with isocyanate-reactive components in the formulation	coils and electric wires
type IV	two-part solvent-borne; one part is the prepolymer polyisocyanate, and the second one contains all other components (polyol(s), catalyst, solvents, pigments, and other additives); ambient or heat curing	plastics, wood furniture, marine exteriors
type V	two-part high solid (>50%) coatings; one part is a prepolymer and the second one is a polyol	leathers, wood, automotive clear coats, refinishes, aircraft, bus, trucks, industrial structure maintenance coatings
type VI	one-component nonreactive low solid (<20%) solvent-borne; high gloss film forms upon solvent evaporation	textiles
powder coatings	one-part reactive system using caprolactam or 1,2,4 triazole blocked aliphatic isocyanates	automotive exterior panels and parts, wires, electrical transmission equipment, surfaces, metal surfaces, outdoor lawn furniture
radiation	high solid coatings, rapid cure, high gloss, not practical for home use or complex shapes; made by reacting isocyanate-capped prepolymer with hydroxyl functionalized acrylate or methacrylate	manufactured wood flooring, cabinets, metal surfaces, plastics
waterborne	broadly applied to one- and two-part systems using aliphatic or aromatic isocyanates; reduces VOC exposure, can be used in hybrid technologies	wood coatings, decorative coatings, artificial leathers, textiles, plastics, inks, architectural, automotive

To avoid viscosity issues, polyurethane adhesives and coatings were initially formulated as two-component reactive systems by mixing low molar mass or oligomeric raw materials, which contained free isocyanates and/or alcohol moieties. Later, the addition of organic solvent was proposed to solve viscosity issues associated with the handling of high-molar mass prepolymers that are required to provide optimal end-use properties.^20^

Nevertheless, environmental concerns and new regulations from the European Union and the United States Environmental Protection Agency, which limit the amount of volatile organic components (VOC) that can be released into the atmosphere, have pushed scientists to explore new approaches to fabricate adhesives and coatings free of VOC and hazardous air pollutants. Thus, waterborne systems have emerged as competitive alternatives to solventborne ones. Waterborne polyurethanes (WPUs) are usually prepared in a similar fashion to solvent-based PUs with the exception that hydrophilic groups are incorporated into the polymer backbone to ensure the dispersion of the chains in water.^21−23^ However, the hydrophilic moieties in WPUs result in a final material with lower water and weather resistance compared to their solvent-based counterparts.^24,25^ Another major drawback is the poor resistance of the WPU coatings toward mechanical strains and high temperatures. To improve the material properties, additional curing is generally applied by incorporating radiation curable species, e.g. epoxides or acrylates, or moisture sensitive groups, e.g. alkoxysilanes. Table 3 summarizes the main advantages and drawbacks of each technology that should be taken into account when designing and processing formulations.

**Table 3:**
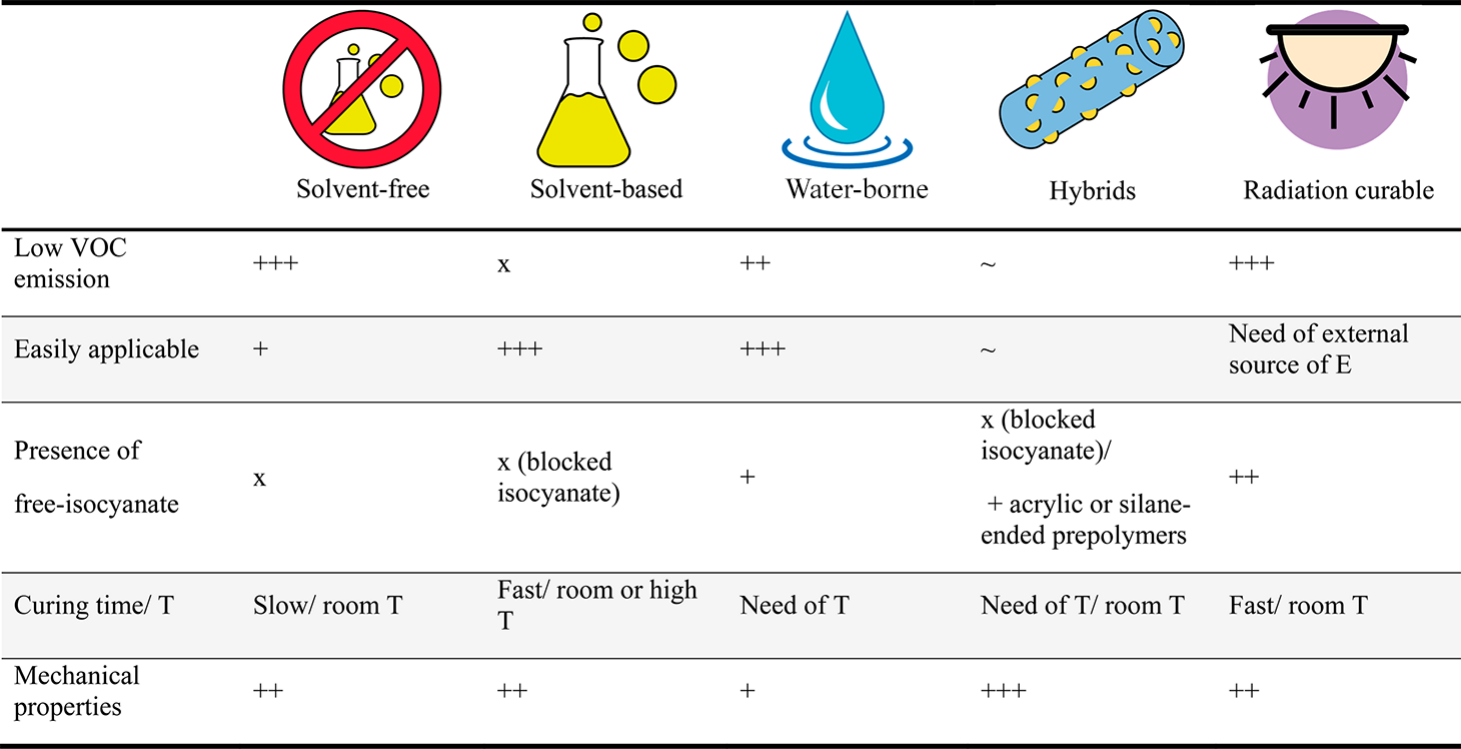
Advantages and Drawbacks for the Different Types of Polyurethane Adhesives and Coatings^19,26−28^^,^^a^

a +++ excellent; ++ good; + slightly good; × poor; ∼ depends on the formulation.

### Tests for Evaluating the Performance of Adhesives

Adhesion is a complex process in which many factors govern the final adhesive performance. The combination of different adhesion theories explains this complex process.^29^ First, the polymer has to present an ideal viscosity to wet the substrate. According to wetting theory, good wettability is related to the surface tension of the adhesives and substrates. Adhesives with low surface tension values wet the substrate surface easily. In addition, the nature, chemistry, and morphology of the substrate substantially affect the adhesion process. According to mechanical theory, adhesion occurs when the adhesive penetrates the pores, cavities, and other surface irregularities on the substrate. On the other hand, some materials such as wood or glass contain hydroxyl groups that can react/interact with the adhesives by the formation of covalent bonds or hydrogen bonding, which increase the adhesion forces. Based on the chemical bonding theory, covalent and ionic bonds provide much greater adhesion values than secondary forces (hydrogen bonding, dipole–dipole, ion–dipole, or London dispersion forces). Thus, nonpolar plastics such as polyethylene are more challenging to coat/glue due to the poor interactions between them and the polar PHUs. A pretreatment of these plastics is often required to increase the polarity at their outer surface, for instance by oxidation by corona treatment.^30^ Whatever the substrate, prior to coating or gluing, removal of dust and degreasing are prerequisites.

**Figure 2:**
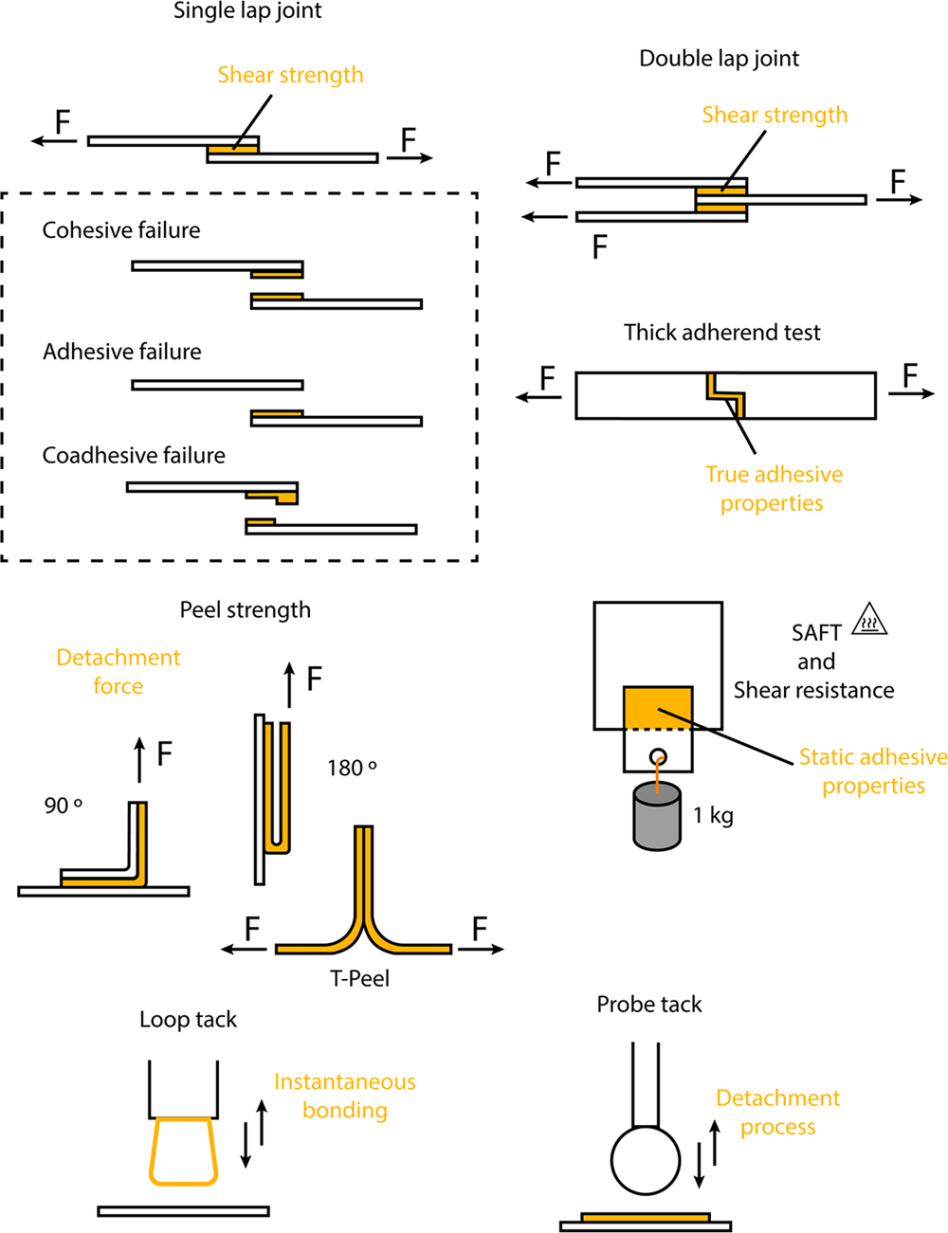
Schematic representation of the evaluation of adhesion for adhesives represented in orange.

In Figure 2, common tests for the quantification of adhesive strength are depicted.

Briefly, lap-shear measurements employing single (ASTM D1002) or double (ASTM D3528) lap joints are the most common tests applied for comparison and quality control of adhesives. These tests determine the maximum shear strength of an adhesive together with providing useful information about the bond failure mechanism (cohesive or adhesive). Structural adhesives present high shear strength values, in the range of 15–30 MPa,^31^ while hot-melt adhesives are characterized by lower shear strengths, usually 3–4 MPa.^32^ Cohesive failure indicates that the maximum strength in the joint is reached, while adhesive failure means the interaction force between the adhesive and the substrate is weaker than the cohesive forces in the polymer.

Evaluation of static loads is carried out through shear resistance and shear adhesion failure temperature (SAFT) measurements (ASTM D4498 can serve as a guide for the preparation of test specimens). Specimens holding 1 kg are evaluated, determining the time or the temperature of failure, respectively. Information about service temperatures and resistance of the adhesive to creep, durability of the joint, are thus provided from these tests. Peel strength measures the required force per area to detach a flexible substrate from a rigid or another flexible one. Typically, rigid structural adhesives present very low peel strength in comparison with flexible adhesives. The test can be carried out with different angles between the adherents, but for T-peel, 180° and 90° are the most common (ASTM D1876 and D3330).

Another important property concerning adhesives, especially pressure sensitive adhesives (PSAs) and hot-melt adhesives, is the tackiness. Loop tack (ASTM D6195) or probe tack (ASTM D2979 or D3121) are the most common procedures to evaluate it. Loop tack is mostly employed for adhesive tapes, whereas probe tack can be performed for a wider range of adhesives.

### Tests for Evaluating the Coating Performance

Evaluation of the performance of NIPU coatings not only focuses on the adhesive performance but also requires measurement of the coating thickness, the appearance (specific gloss, defects), the mechanical properties, and the chemical resistance of the films. Besides the adhesion tests performed for adhesives, some more specific adhesion tests are also used, e.g. the cross-cut adhesion test (ASTM D3359) where the surface removal using a tape is evaluated or the pull off strength test (ASTM D4541) where the perpendicular force that coatings can support on rigid substrates is measured.

**Figure 3:**
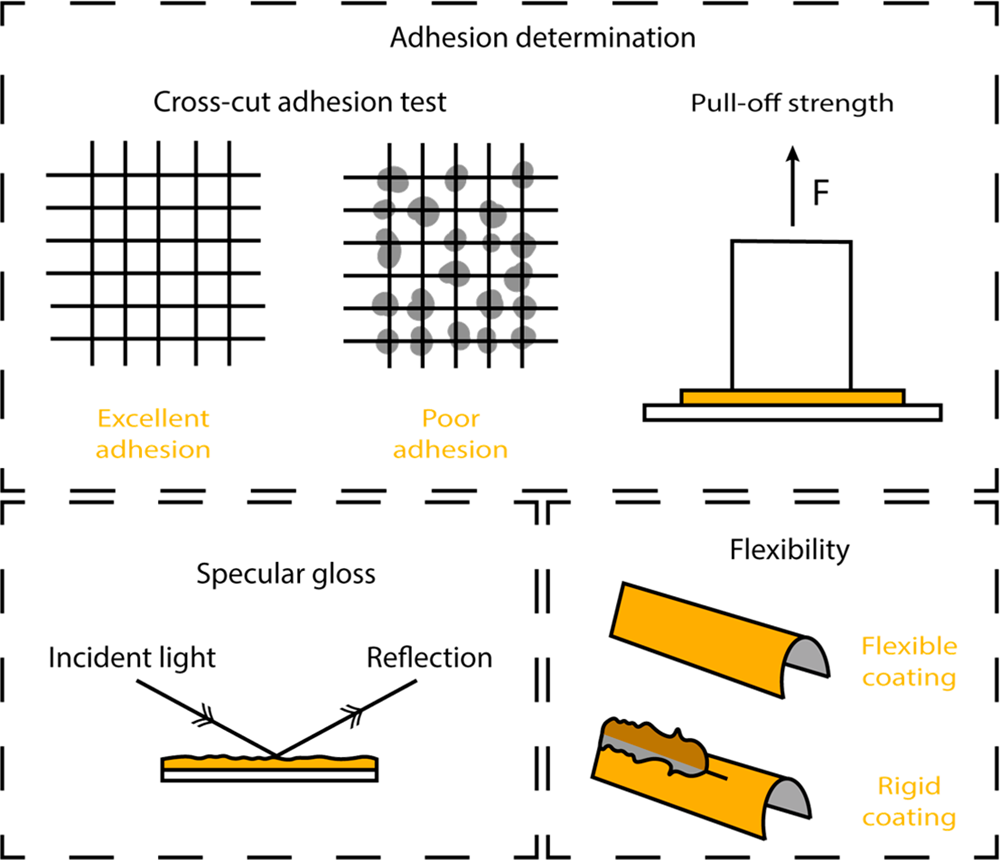
Some of the most common tests for the evaluation of coating performance.

Aesthetic properties are especially important for top coatings. Specular gloss (ASTM D523) classifies coatings in nonmetallic and highly reflective coatings. Protective and high-performance coatings have to meet certain specifications for dry film thickness (ASTM D7091) while wet film thickness measurements (ASTM D4414) can be used to control final dry film thickness. The thickness can be adjusted by the applicator at the time of application. Among the mechanical properties, flexibility (ASTM D522), film hardness (ASTM D3363, by pencil test), abrasion resistance (ASTM D4060, employing taber abrasor), and impact resistance (ASTM D2794) are the most commonly performed tests. Coatings that are used in aggressive environments, such as in the chemical industry, must also offer excellent chemical resistance against different fluids or chemicals such as water (under immersion D870, fog apparatus D1735, or 100% relative humidity D2247), solvents (D5402 through rub test, in which MEK is typically evaluated), and alkali and acidic solutions (ASTM D1308). Anticorrosion properties are crucial for metals exposed to ambient conditions. Boat coatings are an important example of anticorrosive coatings. The salt spray test (ASTM B117) or determining the formation of corrosion between coating and substrate (ASTM D2803) are typical ways to measure anticorrosion properties (Figure 3).

## Approaches for the Preparation of NIPU-Based Adhesives and Coatings

**Scheme 1:**
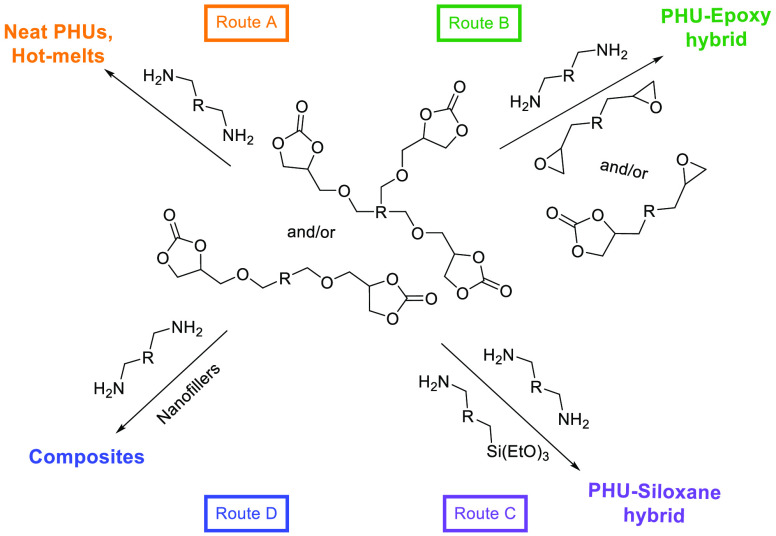
Main (Hybrid) Chemistries Involved for Preparing PHU Adhesives

As PHUs are the most widely used NIPUs for adhesive and coating applications, we will focus on this family of NIPU by describing the various processes used for preparing these materials. We will mainly report PHU materials that have been characterized by at least one of the standard evaluation tests discussed in the PU Adhesives and Coatings: Main Processes and Characterization Tools section. It is important to point out that PHUs differ from conventional PUs by the presence of hydroxyl groups along the polymer chain. While these groups participate in intramolecular and intermolecular hydrogen bonding,^33^ such bonding with the substrate also enhances the adhesion forces. Nevertheless, they disfavor the phase separation present in conventional polyurethanes^34^ and increase the coating/adhesive hydrophilicity with the consequence of enhancing water uptake, which facilitates their wet delamination from the substrate.^35,36^ Various strategies have also been designed to overcome some of the limitations of PHU chemistry (slow reactivity, high temperature, ...) namely by developing orthogonal/hydrid chemistries in combination with the aminolysis of carbonates (Scheme 1).

### PHU Adhesives

Solvent-free formulations are certainly the most attractive route to prepare adhesives as no toxic solvent is released upon evaporation and shrinkage phenomena are limited during the curing process. However, this requires a suitable combination of poly(cyclic carbonate)s to polyamines for the preparation of a viscous formulation that can be applied to the substrate. Cornille et al.^37^ were the first to report the application of neat PHUs as adhesives. Reactive formulations were made of bis- and trifunctional cyclic carbonates blends and (cyclo)aliphatic diamines and subsequently cured by thermal treatment for gluing similar substrates of beech wood, glass, and aluminum (the latter was precovered by an epoxy paint) (Scheme 1, route A). The adhesive performance was evaluated and benchmarked with those of two conventional PUs derived from isocyanates. For glass, the substrate did not resist traction whatever the PHU formulation, underlying that the shear force required to break the adhesive was higher than that needed to break glass. For optimal PHU formulations made of a cycloaliphatic diamine, wood failure also occurred prior breaking the adhesive at a lap-shear strength >15 MPa, while reference PUs only provided adhesion strength of 3–6 MPa. For both supports, the authors postulated that the presence of surface functional groups (silanol for glass or hydroxyl for wood) created additional van der Waals/hydrogen bond interactions with the OH groups of PHUs that were responsible for the excellent adhesion. This hypothesis was further supported by the adhesion values and the predominant adhesive failure mode reported with painted Al. Much lower lap-shear strength values in the range of 2−3 MPa were measured for the various PHU adhesives. Besides, the authors prepared further PU compositions employing diisocyanates and diols with identical backbone structures. Results showed lower lap-shear strength values than those for PHUs when gluing wood, evincing the stronger interaction of PHUs due to hydrogen bonds of hydroxyl groups.

Detrembleur et al.^38^ reported the synthesis of biomimetic PHU adhesives by adding an amino functional catechol, dopamine (DOP), as an adhesion promoter to a PHU formulation made of a trifunctional cyclic carbonate (trimethylolpropane tris-carbonate; TMPTC) and a diamine (hexamethylene diamine, HMDA). At a low DOP loading (3.9 mol %), lap-shear strength values as high as 24 and 28 MPa for Al and wood substrates, respectively, were measured. Interestingly, lap-shear adhesions up to 4.76 MPa were obtained on HDPE. Furthermore, this formulation was also efficient for gluing dissimilar substrates (e.g., Al to SS or plastics) with a similar range of forces between 6.7 and 25.0 MPa. These biomimetic adhesives were found to be competitive to commercial formulations (Teromix-6700 and Araldite2000), provided that the appropriate thermal curing was applied to the PHU formulation.

Hot-melt PHU adhesives are solvent-free systems that are employed by melting thermoplastic polymers on a heated substrate.^39−43^ Tryznowski et al.^39,40^ fabricated hot-melt PHU adhesives for birch wood. Amino telechelic oligoamides made by the condensation of 1,3-diaminopropane (1,3-DAP) with diethyl tartrate^39^ or dimethyl succinate^40^ were chain extended with diglycerol dicarbonate providing PHUs containing polyamide segments. For bonding wood joints, the thermoplastic PHU was melted at 130 °C on both surfaces of the substrates, followed by cooling to solidify the adhesive. This methodology provided moderate adhesion values up to 3.19 MPa. Along the same lines, recent work of Xi et al.^41,42^ has reported the application of saccharide-based NIPUs onto wood joints. Under optimized conditions, pressing the samples at 230 °C for 12 min, sucrose-based NIPUs presented internal bond strength values around 1.02 MPa, 3 times higher than the standard requirement (≥0.35 MPa).^44^

Nair et al.^43^ described the preparation of thermoreversible hot-melt PHU adhesives based on homo- and copolymers of aromatic, bisphenol A-based, and cycloaliphatic, Araldite CY 230-based, bis(cyclic carbonate)s and aminotelechelic oligo(propylene glycol).^43^ Beside the good adhesion values up to 9 MPa on aluminum and 2 MPa on high density polyethylene, the authors showed that the substrates could be debonded manually after thermal treatment up to 100 °C for 0.5 h and then rebonded with no noticeable loss of the shear strength values. Additional examples of hot-melt PHU adhesives may be found in the patent literature and mainly differ by the composition and application/curing temperatures of the thermoplastic.^45−47^

The low reactivity of cyclic carbonates with amines is a major hurdle to the development of PHU adhesives, particularly in applications that require fast curing at room temperature. Therefore, hybrid systems combining PHU with other chemistries (epoxy, sol–gel or composites) have been used to enhance the competitivity of the adhesives (Scheme 1, routes B, C, and D).^33^

Two decades ago, Figovsky et al.^48^ briefly introduced a series of PHU-epoxy hybrid coatings and adhesives using cyclic carbonates, epoxy oligomers, and amine hardeners for potential application in microelectronics. Curing was performed at room temperature for 24 h on Al or SS and the authors claimed a 1.5 to 1.7-fold increase of the lap-shear adhesion strength of the hybrid system onto aluminum and steel (12 and 16.7 MPa, respectively) compared to an epoxy-based adhesive. The adhesive was cured by the combined use of the epoxy resin (by the amine/epoxide reaction) and PHU. The same authors^49^ also reported another PHU-epoxy hybrid adhesive curable at room temperature (for 7 days) by mixing an epoxy resin (DER 331) with carbonated-epoxidized soybean oil (CESBO) and Vestamin TMD as an amine hardener. The shear strength of the hybrid adhesives evolved with the carbonate content and reached maximum of ∼10 and 7 MPa, respectively for carbon steel and aluminum, when the CESBO percentage was increased up to 10 mol %. Similarly, Stroganov et al.^50^ evaluated the adhesive properties of a PHU combined with a BPA-based epoxy resin following Figovsky’s curing protocol (rt, 7 days). They found that the addition of the cyclic carbonate to the formulation improved the lap-shear strength up to 15.8 MPa when room temperature curing was applied. However, a post-treatment of 10 h at 100 °C was required to achieve these values.

Recently, Lambeth et al.^51^ revisited this chemistry to optimize the fabrication of hybrid PHU-epoxy hybrid adhesives by understanding the reactivity of the epoxide and carbonate chemistries. The authors showed that the aminolysis of the epoxide was slightly faster than the cyclic carbonate counterpart, thus forming PHU–epoxy hybrid networks that were fairly uniform with a slight preference for the epoxide ring-opening compared to the cyclic carbonate in the early stage of the adhesive formation. They also showed that the secondary amines formed by aminolysis of epoxides did not contribute to the network construction. They formulated thermoset PHU–epoxy hybrid adhesives for bonding Al plates from trimethylolpropane triglycidyl ether (TMPTGE), trimethylolpropane triglycidyl carbonate (TMPTC), and 4,4′-methylenebis(cyclohexylamine) and evaluated the performance of the adhesives for various compositions cured at 80 °C. Remarkably, the epoxy and hydroxyurethane moieties operated in synergy to create high performance adhesives with a maximum lap-shear adhesion value of 27 MPa for a 50/50 TMPTGE/TMPTC molar composition that is ∼1.7 or ∼1.3 higher than pure epoxy or PHU formulations, respectively. The benefit of merging epoxies chemistries with PHU was further confirmed by Anitha et al.^52^ who introduced hydroxyurethane moieties within amine-cured epoxy systems by utilizing a monofunctional cyclic carbonate additive. At cyclic carbonate content as low as 1–4 mol %, the adhesive performance of epoxy adhesives made of Jeffamine T403 and diglycidyl ether bisphenol A were significantly increased with lap-shear adhesion strength value up to 22 MPa for Al substrate, surpassing the value for the neat epoxy analogue (17 MPa). It has to be noted that many patents have been filed on PHU-epoxy hybrid formulations for (structural) adhesives among other applications.^53−58^

Alkoxysilanes can undergo condensation reaction and cross-link to form a siloxane-linked network by a sol–gel process. They are also able to react with the functional groups at the surface of glass and metallic substrates creating −Si–O–Si– and −Si–O–M– covalent bonds that are beneficial for the adhesive performance.^59−61^ Rossi de Aguiar et al.^59^ combined this sol–gel chemistry with PHUs to prepare hybrid thermoset adhesives. Cyclic carbonate telechelic PDMS (CC-PDMS) was reacted with (3-aminopropyl)triethoxysilane (APTES) as a sol–gel precursor or with blends of APTES and isophorone diamine. After thermal curing at 60 °C, the hybrid adhesives displayed an adhesive strength ranging from 0.9 to 1.3 or 3 MPa for Al-to-glass and glass-to-glass adhesion, respectively. The adhesion was maximum for a composition containing only APTES. Increasing the curing temperature from 60 to 180 °C to favor the sol–gel process was found to be detrimental with respect to the adhesive performance, with a loss of the adhesion strength of ∼50% between 100 and 160 °C and a loss of around 70% at 180 °C. More recently, Gomez-Lopez et al.^60^ engineered a two-step process for the preparation of a monocomponent sol–gel hybrid PHU adhesive. Cyclic carbonate telechelic PHU prepolymers were synthesized by step-growth copolymerization of blends of poly(propylene glycol)- and resorcinol-bis(cyclic carbonate) with hydrophobic Priamine 1074 followed by functionalization of the chain-ends with APTES. The authors illustrated the crucial role of the temperature and the presence of catalysts (HAc, methanesulfonic acid or DBU) on the curing process. While at room temperature, the sol–gel condensation was too slow to synthesize networks within a reasonable time frame, it was significantly accelerated at 100 °C, providing a cross-linked system in less than 1 h. Remarkably, a 5-fold decrease of the gelation time (*t* = 9 min) was measured at 100 °C by the simple addition of 1 wt % of HAc as a condensation catalyst. For the optimal prepolymer composition, lap-shear adhesion values of up to 3 MPa were measured for adhering steel-to-steel. By adapting the PHU composition and incorporating 1.95 mol % of dopamine and APTMS as promoters for adhesion and curing, respectively, the same group presented PHU hybrid systems with adhesion values as high as 21 MPa.^61^

Detrembleur et al. revisited the formulations for gluing Al substrates and highlighted the importance of the thermal curing conditions on the adhesion performance. They showed that the adhesives underwent facile wet delamination due to the hydrophilic nature of PHUs, which favored water absorption.^62^ To limit this delamination, hydrophobic segments (PDMS)^62^ or biorenewable hydrophobic cyclic carbonates (issued from vegetable oils)^63^ were used in the formulations (Scheme 1, route D). By loading the formulations with SiO_2_ or ZnO nanofillers, water uptake was strongly decreased and a remarkable enhancement of the adhesive performance and mechanical properties was noted. These improvements were optimal when using 5 wt % ZnO nanoparticles functionalized by cyclic carbonate groups, with an increase of lap-shear strength from 11.2 to 16.3 MPa when the functionalized ZnO nanofillers were incorporated in the formulation. All these benefits were attributed to the higher cross-linking density of the adhesive that was a result of the aminolysis of the cyclic carbonates at the nanoparticles surface. Interestingly, while composite NIPU adhesives made of native or epoxy-functional ZnO fillers resulted in cohesive failure, formulations made from cyclic carbonate-functional fillers presented an adhesive failure mechanism due to the higher mechanical resistance of the material. Similar formulations were developed by the same group by using carbonated soybean oil as the poly(cyclic carbonate) to design partly biobased composite PHUs (Table 4). High adhesion values up to 12 MPa were obtained for adhesion of Al–Al and SS-SS substrates observing mostly a cohesive failure.^63^

**Table 4:** Summary of the Principal Properties of the NIPU-Based Adhesives Reported in Academia

type of adhesive	substrates	curing conditions	speed of test (mm·min^–1^)	lap-shear strength (MPa)	ref
solvent-free	wood	80 °C, 12 h + 150 °C, 30 min	100	15.0 ± 1.5	(37)
	aluminum (Al)^a^			2.0–3.0	
solvent-free	Al	100 °C, 18 h	2	24.1 ± 1.7	(38)
	stainless steel (SS)			22.1 ± 0.9	
	beech			28 ± 1.7	
	PMMA			17.9 ± 1.3	
	HDPE			4.76 ± 2.5	
	dissimilar substrates			6.7–25.0	
hot-melt	birch wood	130 °C, 1.18 MPa, 30 min	10	0.67^b^	(40)
				3.19^b^	(39)
hot-melt	pine	220 °C, 2.75 MPa, 6 min	2	3.16 ± 0.05	(41)
				3.62 ± 0.02^c^	
				3.38 ± 0.04^d^	
				2.76 ± 0.09	
				1.32 ± 0.08^c^	
				1.24 ± 0.04^d^	
	beech	230 °C, 12 min^e^	f	1.02^g^	(42)
hot-melt	Al	*T* not reported, 24 h	50	9^h^	(43)
	HDPE			<2^h^	
	polyimide			>1.5 kg/cm^i^	
PHU–epoxy hybrid	Al	rt, 24 h	not reported	12	(48)
	steel			16.7	
PHUE–epoxy hybrid	carbon steel	rt, 7 days	5	10	(49)
	Al			7	
PHU–epoxy hybrid	not reported	22 °C, 7 days	not reported	15.8	(50)
		22 °C, 7 days + 100 °C, 10 h		22.8	
PHU–epoxy hybrid	Al 2024-T3	80 °C, 48 h	ASTM D1002	27	(51)
PHU–epoxy hybrid	Al	30 °C, 18 h	10	22	(52)
		+ 80 °C, 1 h			
		+ 100 °C, 2 h			
PHU–siloxane hybrid	glass	60 °C, 24 h	1	3^j^	(59)
PHU–siloxane hybrid	SS	100 °C, 24 h	1	2.9 ± 0.6^k^	(60)
PHU–siloxane hybrid	SS	100 °C, 24 h	1	21.6 ± 0.7	(61)
	Al			20.9 ± 0.9	
	oak wood			12.8 ± 2.4	
	polyamide			2.8 ± 0.7	
	HDPE			0.8 ± 0.2	
	PPMA			1.8 ± 0.2	
composite	Al	70 °C, 12 h	2	16.3 ± 1.4	(62)
	Al, SS	+ 100 °C, 3 h		3.7–11.7	(63)

a Cover of epoxy paint.

b Cohesive forces investigated trough mechanical testing of the NIPU–wood joints.

c 24 h cold water.

d 2 h boiling water.

e Three-stage hot pressing cycle (pressure 33 kg/cm^2^, 4 min; 15 kg/cm^2^, 5 min; 5 kg/cm^2^, 3 min).

f Uniformly load making the specimen damaged within (60 ± 30) s according to China National Standard GB/T 17657-1999.

g Internal bond strength.

h No remarkable differences after thermoreversible adhesion at 100 °C of the materials.

i Peel strength values.

j Kept adhesive performance above the 50% after 10 days at 160 °C.

k Kept adhesive performance after 4 days immersed in water.

### PHU Coatings

PHU coatings share conceptual similarities with the curing chemistry of PHU adhesives. The main difference is the lower viscosity required for the coating formulations as they have to form a homogeneous thin film on large 2D or 3D substrates.^64^ When solvent-free formulations do not fulfill this requirement, solvent-based or water-borne formulations can be used.

### Solvent-Free PHU Coatings

**Scheme 2:**
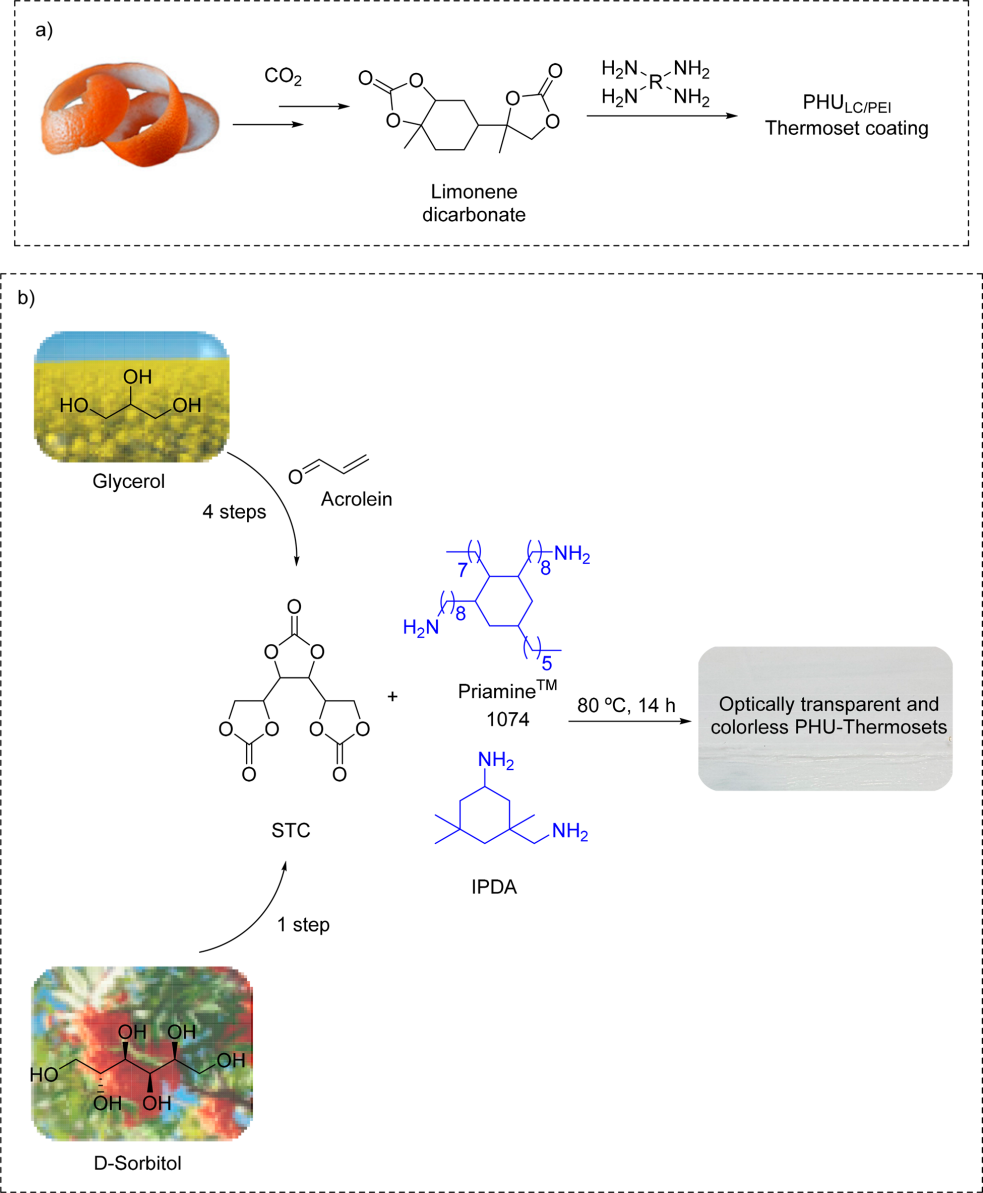
(a) 100% Biobased NIPU Coatings Produced by Melt Phase Polyaddition of Limonene Carbonate and PEI (Thickness of 500 μm) onto a Glass Substrate (Adapted from the Work of Schimpf et al.^65^ Copyright 2017 American Chemical Society) and (b) Synthesis of Sorbitol-Derived PHU for Optically Transparent and Colorless Coatings (Adapted from the Work of Schmidt et al.^66^ Copyright 2016 American Chemical Society)

The handling and processing of molten precursors generally offer a realistic solution to the viscosity constraints. For example, Schimpf et al.^65^ mixed molten limonene dicarbonate and Lupersol (i.e., a polyamine derived from poly(ethylene imine) (PEI)) at 160 °C prior to deposition onto heated glass plates (Scheme 2a) to provide colorless, glossy, and transparent coatings when ultrapure limonene carbonate was used. Unfortunately, no specific coating properties were evaluated in detail.

In order to reduce the temperature required to homogenize the formulation, Schmidt et al.^66^ tailored viscous PHU oligomers by mixing in a three-roll mill sorbitol tris(cyclic carbonate) with Priamine 1074 or a blend of Priamine 1074 and isophorone diamine (Scheme 2b). The resultant coatings were colorless, optically transparent, hydrophobic and scratch resistant. Their film properties varied from highly flexible and soft (*T*_g_ of 29 °C and Young’s modulus of 12 MPa) by using Priamine 1074 as sole amine to stiff (*T*_g_ of 60 °C and Young’s modulus of 630 MPa) when increasing the content of IPDA in the formulation.

The presence of pendant hydroxyl groups in PHUs makes the coatings more hydrophilic than conventional PUs, which might be detrimental for long-term utilization, particularly in wet environments. Some authors have studied the hydrophilicity of PHU coatings derived from viscous cyclic carbonates by determination of the contact angle with water or by equilibrium water absorption measurements.^39−41,67,68^ In order to decrease the hydrophilicity of the coatings, Detrembleur et al.^38,62,63^ added 5 wt % of hydrophobic amino-telechelic PDMS that raised the water contact angle from 48 to 85°. Furthermore, the incorporation of cyclic carbonate functionalized ZnO nanofillers strongly increased the hydrophobicity of the PHU coating, with a water contact angle as high as 114° due to a higher degree of cross-linking that limited the coating water uptake and swelling. Crosscut adhesion tests performed on these PHU coatings deposited on aluminum substrates were classified as 5B, meaning the absence of any coating peeling. In addition, all coatings surpassed 350 MEK double rubs. Substitution of trimethylolpropane triglycidyl carbonate (TMPTC) for carbonated soybean oil (CSBO) as well as the incorporation of aromatic diamines into the formulations also increased the hydrophobic nature of PHU films. Furthermore, all coatings presented excellent dry adhesion (5B) and high solvent resistance to the MEK double rub test (>200).

Fluorinated cyclic carbonates (up to 3 wt %) were added to a PHU formulation by Wu et al.^69^ to increase the hydrophobic behavior of the coatings after curing at 120 °C. These coatings displayed water contact angles of 107° and provided good stain and corrosion resistances to tin substrates.

### Solvent-Based PHU Coatings

**Scheme 3:**
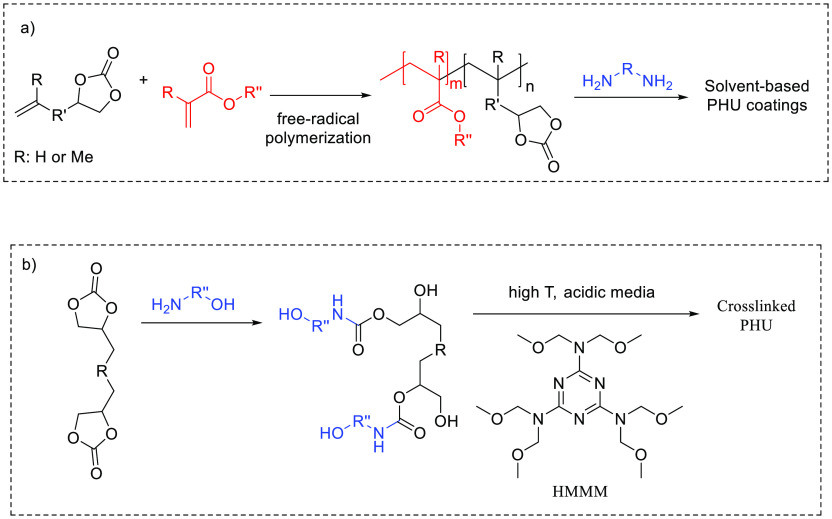
(a) General Strategy for the Preparation of Vinyl-Type Prepolymers by Free-Radical Copolymerization of Vinyl Monomer Mixtures with Some of Them Bearing a Cyclic Carbonate Group and Their Crosslinking through Aminolysis Resulting in PHU Coatings^70−72^ and (b) General Procedure for Producing PHU Solvent-Based Coatings from OH-Terminated Carbamates Cured with HMMM^74,75^

With solid poly(cyclic carbonate)s that do not easily melt at reasonable temperatures the utilization of solvents becomes mandatory. The first solvent-based PHU coatings were reported by Kalinina et al. by exploiting vinyl-type prepolymers bearing pendant cyclic carbonates moieties.^70^ Formulations made of poly[3-(2-vinyloxyethoxy)-1,2-propylene carbonate-*co*-*N*-phenyl-maleimide] and ethylene or hexamethylene diamine cross-linker were prepared in DMF and were cured at 150 °C. Poor substrate adhesion and mechanical resistance of the coating were however noted. By replacing the aliphatic amines by an aromatic one (4,4′-diamino-3,3′-dimethyldiphenylmethane), the authors managed to improve the resistance against chemicals, acids and alkalis; however, the adhesion onto steel and the impact strength remained low. Similarly, Webster et al. synthesized a series of more hydrophobic coatings by curing formulations made of copolymers of vinyl neodecanoate (VV9) or vinyl neononanoate (VV10) and vinyl ethylene carbonate (VEC) with tris(2-aminoethyl)amine or diethylenetriamine in propylene glycol monomethyl ether at 80 °C (Scheme 3a).^71^ Coatings derived from 40/60 w/w [VEC]/[VV9] were glossy and resistant to MEK but displayed poor impact resistance as a result of high cross-linking density.

Recently, Morales-Cerrada et al.^72^ carried out the copolymerization of butyl acrylate, methyl methacrylate, and glycerol carbonate methacrylate for hydroxyurethane-acrylate coatings (Scheme 3a). The copolymers were dissolved in MEK and mixed with tris(2-aminoethyl)amine and cured at 80 °C for 2 h. The cross-linked materials presented high adhesion to stainless and glass. For glass substrate, the authors correlated the adhesion to the formation of hydrogen bonds between PHU and the silanol groups at the glass surface.

Kathalewar et al. investigated the structure-properties relationship of various solvent-based PHU coatings made of bis(cyclic carbonate)s derived from cashew nut shell liquids, containing mainly cardanol analogues, and diamines, i.e., hexamethylene (HMDA) or isophorone diamine (IPDA).^73^ Optimum formulations were obtained from ternary mixtures made of the bis(cyclic carbonate) and a HMDA/IPDA blend as hardening system. High impact resistant coatings were obtained, and the abrasion resistance increased with the content of HMDA. However, the introduction of HMDA within the formulation slightly softened the coating and reduced both its adhesion strength (between 2.83 and 2.37 MPa, adhesive failure) and its scratch hardness compared to PHU systems made with IPDA as sole hardener. The coatings presented good resistance in water, acidic, or alkali media and were found to be resistant above 200 rubs to polar (MEK) and nonpolar (xylene) solvents. The PHU coating performance was benchmarked against those of epoxy analogues and demonstrated better adhesive strength, comparable mechanical properties, and improved chemical resistance due to the presence of the OH groups in the polymer which increased the coating/substrate and the interchain interactions by hydrogen bonding. However, their thermal stability was ∼20 °C lower than that of epoxy coatings.

As a variant, Kathalewar et al.^74^ and Asemani et al.^75^ exploited the OH moieties of a dicarbamate obtained by aminolysis of a bis(cyclic carbonate) with an aminoalcohol to prepare cross-linked coatings by thermal curing with hexamethoxy methylene melamine (HMMM) in the presence of a solvent and acid catalysis (Scheme 3b). Excellent abrasion resistance, impact resistance up to 6 H, and excellent chemical resistance was achieved up to 200 rubs to polar (MEK) and nonpolar (xylene) solvents. They suggested that due to the presence of multiple polar groups—urethane linkages and unreacted OH groups—the interaction between PHU and the metallic substrate was enhanced, considerably reducing the delamination.

In the quest for materials with reduced carbon footprint, approaches that utilize multifunctional biorenewable cyclic carbonates (from pentaerythritol, sucrose soyate, or vegetable oils),^76,77^ that minimize the use of solvents, and that lower processing/curing temperature are highly desirable. As such, a large portfolio of PHU formulations has been reported in the literature. Organocatalyst-driven (DBU or TBD, 1 mol %) thermal curing of cyclic carbonates based on sucrose soyate or soybean oil with tris(2-aminoethyl)amine in ethyl 3-ethoxypropionate/toluene furnished PHU coatings for steel with high crosshatch adhesion (5B), a pencil hardness 2–3 H, and MEK resistance to >400 double rubs. For analogous formulations cured with higher catalyst loadings, the concomitant aminolysis of the ester bonds of sucrose or the vegetable oil induced the formation of some amide linkages which decreased the coatings performance, especially regarding the resistance to MEK. Interestingly, using lithium trifluoromethanesulfonate (LiOTf) in synergy with a superbase at a 1:1 molar ratio enabled shorter curing times (i.e., 45 min instead of 3 h at 120 °C) at a lower temperature (80 °C) without affecting the general characteristics of the coatings, with the exception of a slight decrease in hardness.

PHU can act as a diffusion barrier to oxygen or H^+^ ions making them suitable to design corrosion protective coatings. Pathak et al.^78^ prepared coatings by mixing cyclic carbonates derived from modified castor oil fatty acid and (cyclo)aliphatic or aromatic diamines in a xylene/MEK mixture, and by curing the formulation for 1 h at 140 °C. All coatings displayed 100% adhesion, as measured by the tape adhesion method, pencil hardness higher than H–2H, good flexibility (no visible cracks) as well as good impact (70.86 lbs-in.) and acid resistance (5% HCl) with no blistering or loss of gloss. The authors also established anticorrosion performance/amine hardener structure relationships. While coatings cured with aromatic hardeners were highly rigid and displayed excellent protective barrier to the substrate, systems cured with the aliphatic diamine (HMDA) were less efficient.

The reaction between the cyclic carbonate/amine chemistry has been exploited to design coatings from commercially available polymers such as PDMS or poly(ethylene imine) (PEI) with antibacterial properties.^79−81^ For instance, primary amines of PEI were reacted with a mixture of quaternary ammonium functionalized ethylene carbonate and benzyl-, C_8_- or C_12_- alkyl and/or allyl-bearing five-membered cyclic carbonates. Aminolysis of the cyclic carbonates provided PEI bearing hydroxyurethane bonds and the antibacterial groups (ammonium or benzyl/long alkyl chains). The water insoluble coatings showed a growth inhibition of Gram-positive and Gram-negative bacteria above 95%, which reached as high as 99% when PEI was cross-linked. This cross-linked PEI was obtained by adding a cyclic carbonate bearing an allyl group to the formulation, followed by UV cross-linking in the presence of a photoinitiator. However, leaching out the polymer from the surface was observed, preventing the authors from tailoring highly adherent antibacterial coatings with long lasting properties.

Some solvent based systems have found industrial interest.^82^ For instance, the coating division of BASF^83^ patented the use of biobased hydroxy-urethanes as reactive diluents in solvent-borne automotive coating formulations.

### Water-Borne PHU Coatings

**Scheme 4:**
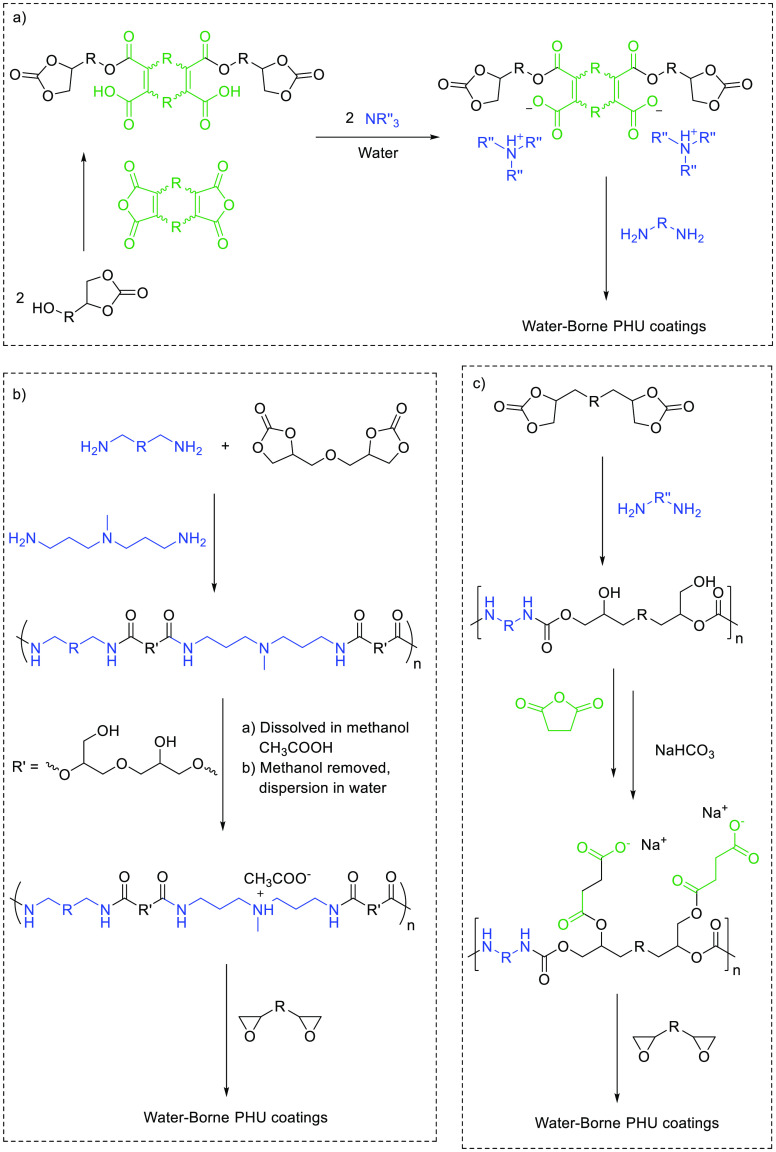
General Strategies Employed for Preparing Water-borne PHU Coatings from (a) Aminolysis of Cyclic Carbonate Based Waterborne Dispersions^87,88^ and (b) Ammonium Bearing PHU Dispersions^89^ and (c) PHU Sodium Carbonate Dispersions Cured with Epoxy Compounds^90^

Water-borne PHU formulations are attractive to surpass the main limitations of the above systems, thus avoiding the use of organic solvent and solving viscosity issues. However, preparing these formulations directly in water is challenging due to the lack of water-soluble carbonated precursors and the occurrence of side-reactions. One of the main side reactions is the hydrolysis of cyclic carbonates that generates unreactive alcohol groups and carbon dioxide. The latter acidifies the reaction medium, leading to some protonation of the amines and therefore may stop the polymerization.^84−86^ Recently, new strategies have emerged that enable the synthesis of water-borne PHU formulations that show promise for coating applications. All these approaches utilize monomers or PHUs that contain carboxylic acid or tertiary amine groups within their structures, such that they can be ionized by adding a base or an acid, respectively. This strategy allows the dispersion of the monomers or polymers in water in the form of latexes able to react with appropriate hardeners (Scheme 4).

Wu et al. fabricated a library of carboxylic acid-functional poly(cyclic carbonate)s either by reacting trimellitic-, pyromellitic-, or benzophenone-3,3′,4,4′-tetra-carboxylic dianhydride with glycerol carbonate or via esterification of partly carbonated sorbitol with maleic anhydride. This furnished water dispersible precursors upon neutralization with 2-dimethylaminoethanol or trimethylamine, respectively (Scheme 4a).^87,88^ The thermal curing of the aqueous dispersion with (cyclo)aliphatic diamines at 90–120 °C provided PHU coatings on glass and tin with hardness of 2B to 3H and crosshatch adhesion grade of 0 (good) to 1 (moderate) and good resistance to impact. All coatings also displayed high gloss and excellent chemical resistance to toluene, xylene, alcohol, or acid; however, they rapidly delaminated in basic conditions.

Zhang et al.^89^ and Ma et al.^90^ synthesized a series of amino-terminated PHUs oligomers (*M*_n_ up to ∼5000 g/mol) containing tertiary amine (Scheme 4b) and carboxylic acid moieties (Scheme 4c). Upon appropriate acid/base treatment, the aqueous polymer dispersions were then mixed with a water emulsion/suspension of epoxy hardeners prior to deposition onto glass or Al. After curing (either for 12 h at room temperature followed by a post-treatment at 120 °C for 12 h or via step-by-step thermal increase from 60 to 160 °C within 6 h), coatings with a 2B to 5H hardness and crosshatch adhesion grade of 1 or 2 were obtained and displayed resistance to MEK of up to 100 double rubs. However, through benchmarking experiments, Ma et al. highlighted lower coating performances for water-borne PHUs compared to analogous solvent-based formulations. Finally, some patent literature also exists in the use of water-based NIPU dispersions as paints.^91−96^

### Radiation-Curable PHU Coatings

Two decades ago, Figovsky et al.^97^ introduced the concept of radiation-curable PHU coatings that has been revisited by others in the past decade. All systems share conceptual similarities, i.e., the polymerization/cross-linking of photoreactive precursors containing both (meth)acrylic moieties and urethanes bonds.

The aminolysis of glycerol carbonate methacrylate with diamines and the condensation of urethane diols (made by ring-opening of ethylene carbonate with various diamines) with itaconic acid or (meth)acrylic anhydride enabled the construction of series of di/poly functional monomers that are easily cross-linked upon UV-light photopolymerization. Among these approaches, Han et al.^98^ reported preliminary coating performance on tin plates. The room temperature cross-linking of methacrylic-functional oligo(ester-urethane)s photoinitiated by 2,2-dimethoxy-2-phenylacetophenone gave flexible coatings (from 0T to 1T, tight bend without gap) with good to excellent adhesion (4B–5B).

Chain-end functionalization of amino-telechelic PHU by acrylic groups, either via Michael addition or aminolysis of glycidylether methacrylate, or the modification of the PHU backbone by reacting pendant OH groups with acryloyl chloride, are other approaches to construct UV-curable PHU formulations for coating applications. Generally, these materials are blended with reactive bis(meth)acrylate urethane diluents, which can be used to adjust the formulation viscosity and the curing conditions.^99−101^ Wang et al.^99^ have provided the characterization and performance of coatings made in this way. They designed new coating formulations by mixing UV-curable α,ω-acrylated polyesters with reactive bismethacrylate urethane diluents of various structures. Whatever the chemical structure of the latter, the pencil hardness of thick films cast on glass and Al evolved from 2B at 10 wt % loading, to HB above 20 wt %. For all systems, the impact resistance was maximum at a diluent concentration of 10 wt % while the MEK resistance only surpassed 200 double rubs at a diluent content above 30 wt %.

**Scheme 5:**
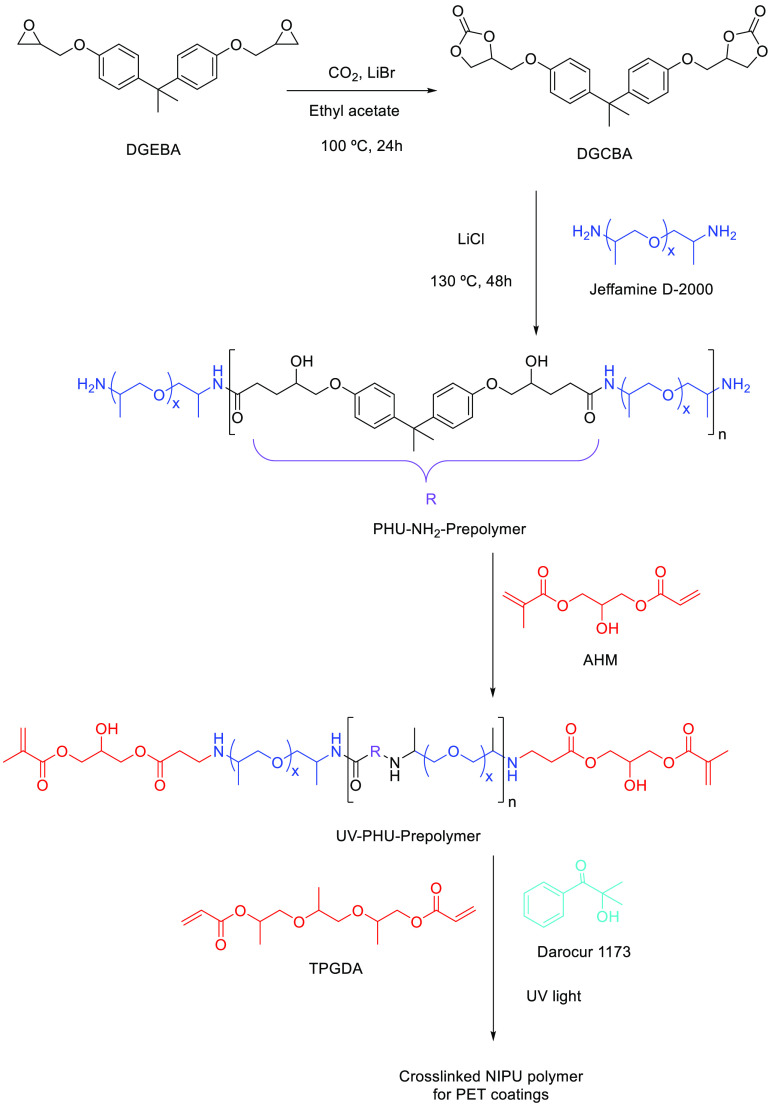
Synthesis of UV-Curable PHU Prepolymers^102^

The utility of these UV-curable systems was highlighted by Hwang et al.^102^ and Zareanshahraki et al.^103^ for covering PET textiles (Scheme 5) or for aerospace applications, respectively. Both approaches utilized (meth)acrylate-terminated PHU prepolymers of various structures, in combination with a reactive diluent (0–5 wt % of tripropylene glycol diacrylate), and were cured with Darocur 1173 or a mixture of Irgacure 184 and 819. The coatings changed the surface properties of PET textiles from hydrophobic to hydrophilic with long lasting durability above 30 washing cycles. The increase of reactive diluent content in the formulations led to lower water absorption values of the PHU materials, which can be correlated to a higher cross-linking density of the PHU network, which limits its swelling. Coatings covering aerospace-grade Al or steel were transparent (with Darocur 1173 as photoinitiator), retained low-temperature flexibility and were resistant to specific chemicals/fluids (aromatic fuel B, hydraulic fluid, lubricating oil, and water), according to military standards.

Uruno et al.^104^ patented coextrusion, coating, and lamination methods for preparing multilayer PHU films. For that purpose, a reactive PHU prepolymer layer bearing unsaturated pendant groups, e.g. (meth)acrylate, was coextruded with other polymer matrices such as poly(vinyl alcohol), polyamide, polyurethane, etc., or coated onto them. The prepolymer was then photopolymerized by employing an UV or electron beam source in the presence of a photoinitiator.

### Hybrid PHU Coatings

**Figure 4:**
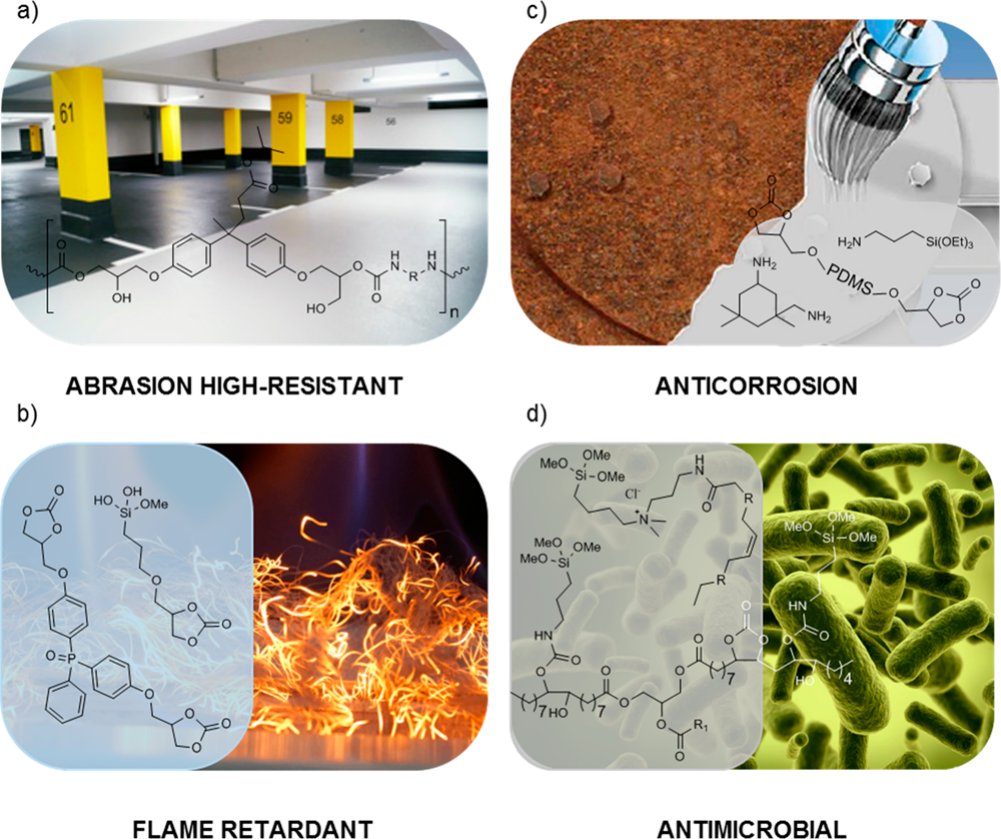
Scope of applications of hybrid PHU coatings with representative components involved in their preparation: (a) abrasion high-resistance coatings based on PHU–epoxy hybrids;^97,105,106^ (b) flame retardant materials based on phosphorus containing PHUs;^107^ (c) anticorrosion coatings based on silica containing hybrid PHUs;^59^ and (d) antimicrobial coatings based on ammonium bearing PHUs.^108^

Hybrid coatings, which combine various chemistries or incorporate additives to the PHU, represent the most widespread technologies within the literature to fabricate PHU coatings. Some of these systems have been employed to confer specific properties, such as abrasion resistance, anticorrosion, flame retardancy, or antimicrobial/bacterial/fungal properties, to the substrate (Figure 4). The most relevant hybrid PHUs systems will be discussed below, and the curing conditions and coatings properties are summarized in Table 5.

**Table 5:** Summary of the Principal Properties of the PHU-Based Coatings Reported in Academia^aa^

type of coating	substrates	curing/application conditions	film hardness	solvent resistance^a^	chemical resistance	cross-cut adhesion	others	ref
solvent-free	glass	160 °C, 16 h					140 GU^j^	(65)
solvent-free	glass	80 °C, 14 h					scratch resistant	(66)
solvent-free	glass	120 °C					19.9–31.2°^k^^,^^l^	(40)
							19.1–32.3°^k^^,^^l^	(39)
							12.5–61.1°^k^^,^^l^	(67)
							58–79°^k^	(68)
solvent-free composite	bare and anodized Al	70 °C, 12 h + 100 °C, 3 h		>350		5B^ASTM^	>85°^k^	(62)
	Al			>200		5B^ASTM^	>95°^k^	(63)
solvent-free	Al	100 °C, 18 h				5B^ASTM^	57–61°^k^	(38)^a^
solvent-free	tin	120 °C, 6 h	H^Pe^			0^ISO^	60^e^	(69)
							504 h^m^	
							>106.8°^k^	
solvent-free	pine	130 °C, 24 h					49.2°^k^	(41)
		300 °C, 5 min				5B^ASTM,^^o^	62.3°^k^	
solvent-based	cold-rolled steel (CRS)	150 °C, 3 h			ac., alk., DMF, eth, acetone	poor		(70)
solvent-based		80 °C, 45 min + rt, 7 days	172^Kö^	>300				(71)
solvent-based	steel, glass	rt, 1 h, 80 °C, 2 h				5B^ASTM^		(72)
solvent-based	mild steel	150 °C, 10–30 min	2H–3H^Pe^	>200^b^	H_2_O, ac., alk.	2.37–2.83^c^	70^d^	(73)
solvent-based	mild steel	150 °C, 5 min	4H^Pe^	>200^b^	ac., alk.	5B^ASTM^,4.9^c^	70^d^	(74)
							>1000^f^	
solvent-based	CSR^g^	150 °C, 30 min + rt, 7 days	3H–6H^Pe^, 160–178^Kö^	>300	H_2_O, DEET, alk., aro. fuel, hy. fl. not to ac.	5B^ASTM^, 2–4B^ASTM,^^h^	fail^i^ 10–30^d^	(75)
solvent-based	tin	100 °C, 30 min	2H^Kö^		poor H_2_O, EtOH, ac., alk.	1^ISO^	<10^e^	(76)
solvent-based	bare steel	120 °C, 3 h	65^Kö^, 2H^Pe^	>400		5B^ASTM^	172^d^	(77)
solvent-based	mild steel	140 °C, 1 h	>H		ac. poor: alk.	5B^ASTM^	71^d^	(78)
							NaCl^t^	
solvent-based	glass	spin-coating					*B. Subtilis*^n^	(79)
							*E. coli*^n^	
solvent-based	glass	rt, 2 h + 60 °C, 16 h					Gram-positive,^n^ Gram-negative^n^	(80, 81)
water-borne	tin	90 °C, 1–2 h + 120 °C, 2 h	HB–3H^Pe,^^p^		xy, to, EtOH, ac. bad to alk.	0–1^ISO^	optimal gloss values	(87)
			H^Pe,^^p^, 0.74^Pen,^^p^			0^ISO^	70^e^	(88)
water-borne hybrid	glass	rt, 12 h + 100 °C, 2 h or rt, 7 days	2B^Pe^, 14^Kö^	>100			>80^e^	(89)
water-borne	Al	60 °C, 2 h + 120 °C, 2 h + 160 °C, 2 h	3H^Pe^			1^ISO^	35–68°^k^	(90)
radiation-curable	tin	UV-cured, rt, 30 min	2H–2B			5B–4B^ASTM^		(98)
radiation-curable	Al	UV-cured + rt, 3 days	2B–HB^Pe^	>200			88–92^e^	(99)
radiation-curable	steel	UV-cured					25.5^Young^	(100, 101)
radiation-curable	polyester textile	UV-cured + 50 °C, 24 h at 75% RH					>30 washing cycles	(102)
radiation-curable	Al 2024-T3	UV-cured, 3 passes^q^		85–90	H_2_O, hy. fl. poor: aro. fuel, lub. oil		–54 °C, passed^i^	(103)
PHU–epoxy hybrid		rt, 5–8 days	2H^Pe^		ac., alk., NaCl^r^	4B^ASTM^	50^e^	(97, 105, 106)
PHU–epoxy hybrid	Al	60 °C, 2 h + 120 °C, 2 h	4H–5H^Pe^			1^ISO^		(90)
PHU–epoxy hybrid	CRS	rt, 7 days		100	aro. fuel, hy. fl., lub. oil	5B^ASTM^	–54 °C, passed^i^	(109)
PHU–nanocomposite hybrid	Al	75 °C, 24 h		190		5B^ASTM^	gloss 70–100^e^	(116)
PHU–nanocomposite hybrid	Al	100 °C, 24 h				1^ISO^	gloss 132–140	(107)
PHU–POSS hybrid	Tin	100 °C, 8–12 h	2H–3H			1^ISO^	50^e^	(117, 118)
PHU–POSS hybrid	glass	80 °C, 14 h + 100 °C, 4 h					scratch resistant^s^	(119)
PHU–silica hybrid	carbon steel	130–140 °C, 3 h			H_2_O, ac. poor: alk.	5B^ASTM^	flame retardant	(120)
PHU–Ly–gibbsite hybrid	stainless Steel	80 °C, 24 h + 100 °C, 4 h					flame retardant	(121)
PHU–MWCNT hybrid	tin	60 °C, 12 h + 90 °C, 4 h	HB			0^ISO^	50^e^	(122)
nanocomposite PHU hybrid	mild steel/Al	70 °C, 30 min + 135 °C, 1 h	4H	>200^2^	ac., alk., boiling H_2_O	5B	70.8^d^, 500 h^v^, 120 h^w^	(123)
nanocomposite PHU hybrid	steel	rt, 24 h + 100 °C, 2 h					55 days^v^^,^^w^	(124)
nanocomposite PHU hybrid	glass	90 °C, 72 h under vacuum	3B–2H^Pe^				UV-weather resistant	(125, 126)
PHU–sol–gel hybrid	glass	60 °C, 24 h				7^c^	alk.^t^, ac.^t^, NaCl^t^	(59)
	stainless steel					1.4^c^		
	Ti_6_Al_4_V					0.7^c^		
PHU–sol–gel hybrid	Ti_6_Al_4_V	60 °C, 24 h				2.2^x^^,^^c^, 3.0^y^^,^^c^	Hank’s solution^t^	(134)
	stainless steel					3.3^x^^,^^c^, 4.0^y^^,^^c^		
PHU–sol–gel hybrid	Al	rt, 24 h + 80 °C, 12 h + 120 °C, 2 h	202^Pe^	>200		2.95^c^	82°^k^	(108)

a MEK rub test.

b Also resistant to xylene.

c Pull-off test, MPa.

d Impact resistance, lbs in.

e Impact resistance, cm/kg.

f Abrasion resistance, in cycles.

g Iron phosphate pretreated.

h After 24 h, immersed in deionized water.

i Mandrel flexibility (1/8 in.);

j Gloss, 60°.

k Contact angle of water.

l Contact angle of iodomethane.

m No rust immersed in 10% NaCl solution.

n Antibacterial activity against.

o Crosscut adhesion performed on steel. Kept performance after washing in hot water.

p Measured on glass.

q 12 ft/min, 0.70 J/cm^2^.

r Saline solution.

s Retained 85% of gloss after 200 double strokes.

t Anticorrosion properties.

u Against xylene.

v NaCl salt-spray test at 35 °C.

w EIS test in 5% NaCl.

x Laser treatment.

y Oxygen plasma treated.

z Antibacterial activity against *Methicillin-resistant S. aureus*, *P. aeruginosa*, and *C. albicans* without toxicity.

aa ac. acidic solution; alk. alkali solution; aro. fuel aromatic fuel; ASTM according to the ASTM D 3359 scale 0B worst–5B best; DEET *N*,*N*-diethyl-*m*-toluamide; DMF dimethyl formamide; eth. petroleum ether; Hank’s solution: physiological pH solution including sodium, potassium, calcium, magnesium and chloride; hy. fl. hydraulic fluid; ISO ISO 2409 scale: 0 best–5 worst; Kö König pendulum hardness (s); lub. oil lubricating oil; MDF: medium density fiberboard; MWCNTs multiwalled carbon nanotubes; Pe pencil hardness; Pen pendulum hardness; ref reference; rt room temperature; Sh Shore A hardness; Ti_6_Al_4_V titanium alloy; to. toluene; xy. xylene, Young Young’s modulus in MPa.

#### PHU–Epoxy Hybrid Coatings

In addition to the benefits on PHU adhesion, Figovsky et al.^97,105,106^ claimed that combining epoxy and PHU chemistry also improved the mechanical properties of the coatings compared to pure epoxy or PHU ones. This was illustrated for epoxy/cyclic carbonate/amine formulations, which delivered clear smooth films after curing for 5 to 8 days at 23 °C with a pencil hardness 2H. The impact resistance increased from 10 to 15 kg/cm for epoxy resins to 50 kg/cm for the hybrid coatings. The introduction of hydroxyurethane segments within the formulation also improved the adhesion of the coatings (from 2 to 3B for the epoxy resin to 4B for PHU) and the abrasion resistance (average weight loss of the film after 1000 cycles was roughly half for PHU) while maintaining identical chemical resistance to acids, bases, and saline solutions. Following this seminal work, Ma et al.^90^ developed biobased acetone-borne hybrid coatings for aluminum by cross-linking PHU oligomers made from carbonates of renewable diphenolic acid and (cyclo)aliphatic diamines, with commercial BPA-based epoxy hardener. The three-step curing procedure (from 60 to 120 °C in 6 h) enabled the formation of films on Al. The inherent rigidity of the aromatic groups of diphenolic acid–based bis(cyclic carbonate) directly reflected on the surface hardness performances with an excellent pencil hardness. Asemani et al.^109^ exploited the reaction of nonisocyanate polyurethane polyamines (PUPAs) with epoxy adducts for the preparation of PHU–epoxy hybrid coatings cast onto pretreated CRS panels. Formulations were cured at ambient temperature for at least 7 days before testing. The authors reported the low temperature (−54 °C) flexibility, 100 double rubs of MEK resistance and tack-free time of more than 20 compositions. PHU–epoxy hybrid coatings are by far the most widely patented systems, which illustrates the relevance of this technology for future commercial applications.^54−57,110−114^ For instance Nanotech Industries commercializes PHU hybrids for coatings and flooring applications under the trade name of Green Polyurethanes.^115^

#### PHU–Nanocomposite Hybrid Coatings

As in the adhesive field, fillers have been added to improve the performance of PHU coatings. Turunc et al.^116^ prepared reinforced coatings by adding cyclic carbonate functional SiO_2_ nanoparticles (up to 4 wt %) to a PHU formulation composed of carbonated soybean oils and butanediamine in ethanol as solvent containing PDMS as wetting agent and pyridine as catalyst. The adhesion performance of the nanocomposite coating was evaluated on an Al substrate. The surface remained intact upon impact tests and all formulations gave rise to a 5B cross-cut adhesion. The benefit of introducing nanofillers was further demonstrated by the MEK rub test, which showed an increase from 200 rubs for a neat CSBO formulation to more than 400 rubs in the presence of 4 wt % of the filler. Nevertheless, the incorporation of fillers within the PHU coatings had a detrimental effect on gloss that progressively decreased due to some phase separation between the hydrophobic coatings and the hydrophilic filler. To prevent the poor dispersion of the silica within the CSBO-based PHU resin and generate surfaces with high gloss, hydrophilic poly(propylene glycol)-bis(cyclic carbonate) (PPGbisCC) was added to the formulation (at a 50/50 [CSBO]/[PPGbisCC] weight ratio). In that case elongation at break and gloss of the coatings increased. Nevertheless, these PHU coatings were found less resistant to solvent, with MEK double rub resistance between 100 and 190.

Silica fillers may provide thermally insulating chars layers when burning polymers loaded by silica and can also act as diffusion barriers to combustible gases. This feature was exploited by Hosgor et al.^107^ to design flame retardant PHU coatings (Figure 4b). Whatever the formulation, all coatings were found glossy (132–140 at 60°), resistant to impact (no damage), and showed an excellent cross-cut adhesion grade of 1. The authors evaluated the flame retardancy properties by determining the residual char content after thermal degradation and found that it was higher (∼9% of solid residue) when the silica was present in the formulation at 20 wt %.

Polyhedral oligomeric silsesquioxane materials (POSS)^117−119^ have also attracted considerable attention to improve the thermal and mechanical properties of the coating and adhesion performance of various resins. Liu et al. prepared a series of biobased PHU thermosets and nanocomposite PHU/POSS coatings from rosin^117^ or gallic^118^ acid–based cyclic carbonates. Addition of 20 wt % of cyclic carbonate functional POSS within the PHU formulations increased the cross-linking density and consequently the scratch resistance of the coating.^119^

Some other hybrid PHUs have been prepared by combining different chemistries or compounds depending on the final applications of the coatings including ZrO_2_@SiO_2_,^120^ γ-Al(OH)_3_,^121^ multiwalled carbon nanotubes (MWCNTs),^122^ ZnO,^123^ or tetraethyl orthosilicate.^124^ The dispersion of these fillers within PHU can substantially improve the flame retardancy,^120,121^ the mechanical properties,^122^ anticorrosion performances,^123^ or the UV-weathering^125,126^ resistance of the related coatings even at relatively low concentrations (0.5–5 wt %). The use of PHUs in synergy with fillers has also been covered in the patent literature for enhancing the properties of conventional PHUs.^104,127−133^

#### PHU–Sol–Gel Hybrid Coatings

Hybrid coatings combining sol–gel chemistry and PHUs have been engineered to obtain anticorrosion^59,124,134^ and antibacterial/fungal surfaces.^93−95,116^ Zhang et al.^124^ introduced tetraethyl orthosilicate (TEOS) within amino-telechelic PHU/bisphenol A epoxide reactive formulations and prepared protective coatings on steel. The curing procedure (overnight cure at room temperature before postcuring for 2 h at 100 °C) enabled the formation of a PHU–epoxy hybrid network containing inorganic silica domains, thus limiting the penetration of corrosive ions and water within the coating. The resistance of coated steel surfaces to corrosion following immersion in saline medium for 55 days was highest at a TEOS content of 5 wt %. Higher TEOS loadings weakened the protective performances of the layer as a result of microphase separation of the PHU and the inorganic phase.

Rossi de Aguiar et al.^59,134^ improved both the hydrophobic and flexible nature of diglycidylcarbonate PDMS oligomers with a sol–gel process by utilizing reactive (3-aminopropyl)triethoxysilane to construct corrosion resistant PHU–sol–gel hybrid coatings in 20–40 min at 70 °C (Figure 4c). The protective composite layer acted as a diffusion barrier against corrosive agents. The anticorrosion performance was strongly improved by adding phosphotungstic acid (up to 55 wt %) into the formulation. This acid not only catalyzed the sol–gel reaction, but it also provided tungstate anions that are corrosion inhibitors.^40^ On the other hand, coatings for biomedical grade metal were prepared on titanium alloy (Ti6Al4 V) and stainless steel (SS316L).^134^ Adhesion was improved by applying pulsed Nd:YAG laser and oxygen plasma on the substrates. Furthermore, APTES- as well as IPDA-based coatings presented a lower current density (*J*_corr_) than noncoated titanium alloy, demonstrating the protection of the metal against corrosion.

Gharibi et al.^108^ designed bioactive coatings by sol–gel hydrolysis/condensation reactions of two reactive siloxane precursors derived from (1) a carbamate functionalized soybean oil bearing trimethoxysilane moieties and (2) a fatty amide molecule bearing, in addition to the alkoxysilane units, a quaternary ammonium salt (Figure 4d). Curing was conducted under atmospheric moisture conditions. Together with excellent MEK resistance (>200 double rubs), an adhesion strength of 2.25–2.95 MPa on Al and good hardness, the coatings presented excellent bactericidal and fungicidal activities against selected microorganisms—*Methicillin-resistant S. aureus* (MRSA), *P. aeruginosa*, and *C. albicans*—while remaining cytocompatible. The ammonium groups were responsible for the bactericidal and fungicidal activities.

Similar chemistry has been employed in the literature to patent PHU–silica hybrid systems as coatings with good barrier properties.^133^

## Conclusions and Outlook

Having been developed over the course of several decades, polyurethanes (PUs) have become one of the most widely used classes of polymers and today are found in many high-performance materials, such as foams, thermoplastics, coatings, and adhesives. Nonetheless, guided by environmental concerns and legal obligations, greener and safer alternatives to conventional PUs are now being sought in order to avoid the use of toxic isocyanates that are one of the primary components used in their formulation. Poly(hydroxyurethane)s (PHUs), formed by the polyaddition of poly(amine)s and poly(cyclic carbonate)s, have emerged as the most appealing and versatile non-isocyanate polyurethanes (NIPUs) for coating and adhesive applications. This review has shown that some formulations can now provide materials with competitive performance as compared to their conventional PU counterparts. However, we should emphasize that the performance of many newly developed products in this field are not benchmarked to those of commercial PU analogues, which makes direct comparison difficult. We strongly encourage researchers to perform such benchmarking studies in their work.

Some major obstacles still exist to translate the academic work that has been done on PHUs to marketable and viable industrial products. The first and most important aspect is associated with the curing conditions that have to be applied to reach materials of comparable performance. Indeed, unlike the alcoholysis of isocyanates, the aminolysis of five-membered cyclic carbonates is slow and the curing of PHU formulations has to be performed by thermal treatment (60–120 °C). In contrast, room temperature can be used for most conventional PUs. Recent studies have shown that combining PHU chemistry with complementary reactive groups, i.e., epoxy, sol–gel, radiation-curable, and/or adding appropriate nanofillers (that can be grafted to the PHU matrix) can facilitate the curing of the formulations and improve the properties of the final material. Nevertheless, even if some curing can now be done at room temperature, reaction times are still too long, which does not facilitate the implementation of the technology in the most relevant applications. When thermal curing can be tolerated, some of the previous systems are of great promise. Future research directions to overcome the slow curing process should explore the utilization of cyclic carbonates that can be more easily aminolyzed at room temperature, e.g. larger seven- or eight-membered cyclic carbonates^135−137^ or activated five-membered ones such as the emergent exovinylene cyclic carbonates.^138−140^ Despite their greater reactivity, their production has to be optimized to furnish formulations that are cost-effective and price competitive with respect to conventional PUs, which is far from trivial. Indeed, cost is certainly the second major limitation that is preventing the utilization of non-isocyanate polyurethanes at the industrial scale. This is primarily the result of the limited availability of poly(cyclic carbonate)s. While many poly(amine)s are commercially available at large scale and low cost (notably those used as hardeners for epoxy resins), poly(cyclic carbonate)s are not accessible in large volumes when compared to isocyanates and are therefore less cost-effective. On the other hand, these cyclic carbonates are easily accessible by multiple chemistries, notably by coupling CO_2_ to epoxides (that can be obtained by the transformation of bioresources, e.g. vegetable oils). Therefore, optimized synthesis pathways are required to produce the large range of poly(cyclic carbonate)s needed for pushing forward the use of PHUs in real applications. Currently, this technology is still more expensive than that of PUs, although it should be noted that the latter has been optimized for decades to reduce costs as much as possible. Financial incentives, i.e., financial support to NIPU technology combined with increased taxes on the use of PUs, coupled with stricter regulations regarding the use of toxic isocyanates should contribute to accelerating the transition of this technology to more sustainable NIPU-based products.
